# Occurrence, treatment protocols, and outcomes of colic in horses within Nairobi County, Kenya

**DOI:** 10.14202/vetworld.2017.1255-1263

**Published:** 2017-10-22

**Authors:** Anderson Gitari, James Nguhiu, Vijay Varma, Eddy Mogoa

**Affiliations:** Department of Clinical Studies, Faculty of Veterinary Medicine, College of Agriculture and Veterinary Sciences, University of Nairobi, Nairobi, Kenya

**Keywords:** pain management, colic, incidence, Nairobi, treatment

## Abstract

**Aim:**

The aim of this study was to determine the treatments and their outcomes in horses with colic in Nairobi County, Kenya.

**Materials and Methods:**

This is a retrospective study to determine the occurrence, treatments, pain management, and outcomes of colic in horses in Nairobi County. Association between pain management protocols and the outcomes of colic with regard to recovery or death was also determined. Data collected from four equine practitioners were organized manually and given numerical codes as appropriate to facilitate entry into the computer. The coded data were entered into Microsoft Excel 2010 and exported to StatPlus pro 5.9.8 statistical package for analysis. Simple association tests were done between various factors and occurrence of colic.

**Results:**

The incidence of colic for the 11 years was 3.1%, which constituted 68.0% spasmodic colic, 27.8% impaction colic, and 4.2% displacement colic. Flunixin meglumine as a nonsteroidal anti-inflammatory drug (NSAID) was used as the only pain management treatment in 85.3% of the cases, flunixin meglumine and butorphanol as NSAID-OPIOD combination in 6.4% of the cases, while buscopan as an antispasmodic was recorded in 5.9% of the cases mainly in spasmodic colic. Univariate analysis revealed simple association between various factors and the type of colic a horse was having. There was an association between the type of colic and the decision-making on the pain management protocol to use, whether single analgesic protocol (*χ*^2^=22.5, p<0.001) or use of analgesic combinations (*χ*^2^=18.3, p<0.001). The type of colic strongly influenced the decision for performing nasogastric intubation (*χ*^2^=265, p<0.001), but performing nasogastric intubation was weakly (*χ*^2^=4.9, p=0.03) associated with horse recovery from colic. Type of colic also strongly influenced the need for the use of metabolic stimulants, particularly vitamin B-complex (*χ*^2^=99.3, p<0.001). Recovery or death of the horse from colic was strongly associated with the type of colic (*χ*^2^=250, p<0.001). The possibility of recurrence of colic was weakly (*χ*^2^=4.6, p=0.04) determined by the type of colic, a horse had. Multivariate logistic regression revealed that the main cause of death was intestinal displacement and the majority of the horses with intestinal displacement died (β-estimate 2.7, odds ratio=0.07, p=0.007) compared to horses that had impaction colic.

**Conclusion:**

The incidence of colic is 3.1%, and the most common type of colic is spasmodic followed by impaction. The most common pain management protocol for colic is NSAIDs, mainly flunixin meglumine, followed by flunixin-butorphanol combination. Surgery for horses with colic in Nairobi County is not commonly done due to impeding poor prognoses. The horse owners tend to prefer euthanasia for such cases.

## Introduction

Colic is a general term that implies abdominal pain and is one of the major causes of death in horses [1]. It most commonly affects the horse among the domestic animals. Colic results mainly from conditions affecting the gastrointestinal tract but occasionally could be due to conditions involving organs of other systems within the abdominal cavity. The anatomical disposition of the gastrointestinal tract of the horse, the nature of digestion, and management practices imposed by man are among the main multifactorial predisposing factors for colic [2].

The key principles in the treatment of horses for colic are pain management, decompression of the gastrointestinal tract, correcting biochemical and fluid imbalances, stimulation and maintenance of gastrointestinal motility as well as reduction of gastrointestinal inflammation. Where pain is severe and non-responsive to medical treatment, immediate surgical intervention is indicated [3]. In addition to pain, other significant indicators of critical cases of colic include heart rate, gastrointestinal borborygmi and signs of hypovolemia. Therefore, careful assessment of these indicators is critical when a horse is presented with colic [4].

Equine colic episodes will often resolve spontaneously or after medical treatment. However, colic emanating from impaction and/or intestinal displacements, especially with strangulation, is invariably fatal if surgical intervention is not done [5]. For example, the decision whether to manage ileal impaction in horses by meticulous medical therapy or early surgical intervention may be difficult. Clinical and physical examination parameters such as severity of abdominal pain, heart rate, presence or absence of borborygmi, and transrectal examination were evaluated on horses at admission and were found not helpful in determining whether or not medical management or exploratory celiotomy would lead to successful resolution of the impaction [6]. Ultrasonography can be a specific and moderately sensitive test for diagnosis of the right dorsal displacement of the large colon (RDDLC) and/or colon volvulus that is 180° [7]. Colic surgery is expensive and prognosis ranges from guarded to good [8].

The most commonly used nonsteroidal anti-inflammatory drugs (NSAIDs) for pain management in horses with colic are flunixin meglumine, phenylbutazone, meloxicam, and ketoprofen with varying levels of effectiveness [9,10].

Alpha-2 adrenergic agonists such as xylazine, romifidine, and detomidine are sedatives that have both analgesic and muscle relaxant effects, and hence have been useful for control of abdominal pain in horses. Analgesia by alpha-2 adrenergic agonists is through stimulation of central alpha-2 adrenoreceptors, which modulates the release of norepinephrine and directly inhibits neuronal firing. This not only causes sedation and analgesia to relieve pain in horses with colic, but also has adverse effects such as bradycardia. It has a profound effect to give relief from both somatic and visceral pain caused by distension or strangulation [11,12]. Detomidine also reduces intestinal motility, which could obscure signs of pain, making it difficult for the clinician to diagnose the underlying cause of the colic. Since detomidine is a potent drug, continued signs of colic 30-60 min after its administration may suggest severity of the underlying cause, thus necessitating surgical intervention. Additional dosages can be titrated in 5-10 µg/kg body weight (BW) increments [12].

Butorphanol, which is a partial agonist and antagonist opioid provide the best pain relief with least adverse effects. It can be used alone or in combination with xylazine or detomidine. The dosage range is 0.05-0.1 mg/kg BW depending on the severity of colic [3].

Spasmolytic drugs provide analgesia by reducing spasms of the intestine. Hyoscine-N-butylbromide is an anticholinergic that acts centrally and peripherally. It is a widely used spasmolytic for management of abdominal pain in horses but has a shorter duration of pharmacological effect on the intestine relative to other anticholinergics such as atropine [13]. Combination of hyoscine N-butylbromide and para-aminophenol derivative (dipyrone) has been used in the treatment of spasmodic colic as well as impaction colic [14].

Decompressing of distended stomach or intestines helps in reducing the intensity of abdominal pain. Nasogastric intubation helps to relieve gastric tympany or remove gastrointestinal reflux that is due to small intestinal obstruction or ileus. The nasogastric tube can be left in place for passive decompression after surgery or in horses with proximal enteritis, but such a case should be checked every 2-3 h to ensure that the fluid effectively flows through the tube [15].

The laxatives mostly used in the treatment of impactions include mineral oil, dioctyl sodium sulfosuccinate, and magnesium sulfate (Epsom salt). Laxatives should never be administered to horses with signs of proximal gastrointestinal obstruction because they remain in the stomach for a prolonged period of time and never get to the obstruction site. This causes more distention of the stomach with subsequent increase in severity of the abdominal pain. It is imperative to be certain of the insertion of the tube into the esophagus to guard against accidental entry of mineral oil into the lungs, which has happened even with skilled equine practitioners, and could be fatal [16]. A study to compare the effects of enteral administration of magnesium sulfate (MgSO_4_), psyllium mucilloid (psyllium) and the combination of MgSO_4_ and psyllium on accumulations of sand in the large colon of adult horses was done. It was found that combined administration of psyllium and MgSO_4_ through nasogastric tube once a day for 4 days consecutively was more effective treatment in resolving the impaction than either of them alone [17].

Fluid therapy should be considered in the treatment of hypovolemia in horses with impactions that are unresponsive to analgesics and laxatives. Fluids can be administered either orally or intravenously [18].

Lidocaine therapy has been proposed as a treatment option for horses with inflammatory conditions of the gastrointestinal tract including post-operative ileus and recovery from ischemic injury [19]. Systemically administered lidocaine has been found to have anti-inflammatory properties such as reduction of mucosal cyclooxygenase-2 expression and neutrophil count in ischemic-injured equine intestine or amelioration of the negative effects of flunixin meglumine on the recovery of injured mucosa [20]. It has been observed that there were no effects of prolonged lidocaine (continuous rate) infusion on gastrointestinal transit, except when in combination with butorphanol [21].

Determination on whether a horse with colic requires surgery should be made promptly. However, not many owners are aware that early surgical intervention could improve the outcome of colic. Owners’ consent for surgery was mostly influenced by the age of the horse, level of fitness for the horse to travel to the hospital, and the cost of surgery [22]. Many veterinarians have used the response to analgesic treatment as an effective way to determine colic cases that require surgery [23]. Indications for exploratory celiotomy will basically include severity of abdominal pain, which is unresponsive to analgesics, deterioration of the cardiovascular status, and abnormalities in peritoneal fluid that are indicative of bowel circulatory compromise [12]. The factors that will influence surgical outcome include single or multiple sites of impaction, the duration of impaction before surgical intervention, size of fecalith, any concurrent displacement or torsion of the intestine and occurrence of post-operative complications [24].

Enterotomy is indicated in impaction colic or presence of enteroliths. Early intervention through jejunal enterotomy leads to a reduction of tissue trauma with quick resolution of the extensive impaction, which may contribute to reduced post-operative complications [25]. Complications of colostomy are rare, but the most common are hemorrhage from the incision edges which can be severe enough to cause melena and hemorrhagic shock [26]. Colopexy is done to prevent recurrence of displacement and large colon volvulus; and colostomy is done in cases of rectal injury as bypass to the rectum. Hernia repair is indicated for inguinal and diaphragmatic hernias that result in colic [12].

The most common reasons for euthanasia of horses with colic without attempting surgical treatment are the financial limitations that make owners unable to pay as well as poor prognosis for the colic. Some horses are euthanized because of the tendency for recurrent colic episodes. The age of the horse is also another reason why owners opt for euthanasia rather than surgical intervention [27].

Some prognostic indicators have been used with some degree of predictability for the survival of colic cases after surgery. The most notable prognostic indicators include heart rate, packed cell volume, plasma lactate, creatinine, and blood glucose concentrations [28]. These indicators are related to the extent, degree and duration of intestinal damage as well as the level of pain and the patient’s cardiovascular status. Horses manifesting colic with elevated plasma lactate levels have poor prognosis than those with normal or low levels. When all factors indicate a poor prognosis, then the most rational and logical decision is to euthanize the horse [29]. Significantly low levels of total proteins, especially albumin and globulin, were seen in horses that died compared to those that recovered from colic in Nairobi [30]. A study to evaluate diagnostic markers in horses with colic found that acute phase protein serum Amyloid A improved the ability to differentiate horses with acute inflammatory colic requiring medical treatment from those that require surgery [31]. The greater response of acute phase proteins seen was in horses with bacterial infections (peritonitis or colitis) than those with strangulating lesions. This is due to the gradual development of the disease process with bacterial infections, hence greater responses of the acute phase proteins [32]. These prognostic indicators influenced the decision for euthanasia or surgical treatment. Therefore, precise and timely diagnosis is important to provide an accurate prognosis, which occasionally may be difficult in some cases of colic until after surgery is performed [33].

The significance of this study was to compile and document data on how colic in horses has been managed in Kenya with a view of publishing recommendations to improve the expected outcomes for various forms of colic. This has not been documented before with reference to the equine veterinary practices in Kenya, which is a developing country with limited resources both for establishing modern state of the art veterinary clinics and for payment of veterinary professional and services fees.

The aim of the study was to determine the incidence, treatment and outcomes of colic in horses in Kenya. The incidence was in reference to the overall occurrence of colic as well as the frequencies of occurrence of the specific types of colic. The treatment was in reference to the various protocols employed by the equine practitioners in Nairobi County, Kenya and the resulting outcome for each protocol and each type of colic.

## Materials and Methods

### Ethical approval

This project was approved by the Biosafety, Animal Use and Ethics Committee of the Faculty of Veterinary Medicine, University of Nairobi. The reference approval number is REF: FVM BAUEC/2015/86.

### Study area

This study was conducted in Nairobi County, Kenya, which is located at 1°17′S 36°49′E and has a daily temperature range between 16°C and 30°C. The horse population in the County is approximately 3,200. The area was purposively selected due to its relatively high population of horses and also the fact that the Jockey and Polo clubs of Kenya are within the County, hence the convenience of carrying out the study in this part of Kenya. Nairobi County has more equine practitioners compared to other counties in Kenya. These factors made it possible to obtain the required sample size for the study.

### Study design and data collection

This was a retrospective study covering 11 years (January 2004-December 2014). Data were collected from 4 equine practitioners duly registered by Kenya Veterinary Board and whose main practice activities are in Nairobi County. The required study data were retrieved from records in these equine practices. It included case identification particulars, dates of colic occurrences, clinical signs, the specific diagnosis, as well as definitive and supportive treatments for each case. Other data retrieved from the records included laboratory specimens taken and their results, diagnostic aids used, responses to treatment, occurrences of recurrent episodes of colic, prognosis, and outcome of each case. Data were recorded in data collection sheets.

### Data management and analysis

The data collected from the retrospective study were organized manually and given appropriate numerical codes to facilitate entry into the computer. The coded data were entered into Microsoft Excel 2010. The data were exported to StatPlus pro 5.9.8 statistical package for analysis. Descriptive statistics were then computed, and the data analyzed for incidence of colic, types of colic (in percentage), analgesics used for pain management in the treatment of colic and the outcomes of colic including mortality (in percentages). Incidence was calculated as the number of horses with colic (numerator) divided by the total number of horses seen by the veterinarians (denominator). Mortality was calculated as the number of horses that died from (numerator) and divided by the total number of horses with colic (denominator).

Simple association tests were done between various factors and the occurrence of colic. Examples of these associations were between the type of colic and treatment, between type of colic and outcome, between type of colic and recurrence, as well as between the treatment and outcome of colic. The associations were determined by Chi-square statistic and considered statistically significant at p<0.05.

## Results

### Descriptive statistics for horses with colic

A total of 4 Veterinary Practitioners in Nairobi County had records from which cases of colic in horses could be retrieved. From the records kept by these 4 practitioners, 12,077 cases of horses had been treated for various diseases from January 2004 to December 2014 among which 3.1% (n=375) had been treated for colic over the 11-year period. Among these 375 cases of colic, 68.0% (n=255) had spasmodic colic, 27.7% (n=104) had impaction colic, and 4.3% (n=16) had colic resulting from intestinal displacements (Table-1). From the records, 75.0% (12/16) of the cases with intestinal displacements had intestinal volvulus, and 12.5% (2/16) had right dorsal displacement of the large colon (RDDLC). The other two types of intestinal displacements were left dorsal displacement of the large colon and intestinal strangulation caused by a lipoma (Table-2). All the horses with intestinal displacements died and the records indicated that diagnoses were made at postmortem. The overall mortality rate among all the 375 cases of colic was 5.6% (n=21).

**Table-1:** Percentages of horses with each type of colic among the 375 cases as recorded in four Equine Practices from January 2004 to December 2014 in Nairobi County, Kenya.

Types of colic	Number of horses with each type of colic (n=375)	Percentage (%) of horses with each type of colic
Spasmodic colic	255	68.0
Impaction colic	104	27.7
Intestinal displacements	16	4.3
Total	375	100

**Table-2:** Percentages of horses with each type of intestinal displacement among the 16 cases of displacement colic as recorded in four Equine Practices from January 2004 to December 2014 in Nairobi County, Kenya.

Types of intestinal displacements	Number of horses with each type of intestinal displacement (n=16)	Percentage (%) of horses with each type of intestinal displacement
Volvulus	12	75
Right dorsal displacement	2	12.5
Left dorsal displacement	1	6.3
Strangulating lipoma	1	6.3
Total	16	100

### Types and use of analgesics in horses with colic

Pain management for horses with colic was done using either single or combinations of analgesics. The most common were the use of single NSAID in 85.3% (n=320) of the 375 colic cases, which was mainly flunixin meglumine in 81.9% (n=307) of the cases and ketoprofen in 3.7% (n=14) of the cases. Combination of NSAIDs and opioids to manage pain was used only in 6.4% (n = 24) of the horses with colic, which mainly included flunixin meglumine and butorphanol. Spasmolytics were used in 5.9% (n=22) of the cases and the one invariably used was buscopan and only in spasmodic colic. Other pain management protocols had less than 1% frequencies of use as shown in Tables-3 and 4. Records indicated that the use of ketoprofen in these Nairobi County equine practices begun in 2014.

**Table-3:** Frequencies for the use of various categories of analgesics and drugs for pain management in horses with colic as recorded for horses treated in four Equine Practices from January 2004 to December 2014 in Nairobi County, Kenya.

Categories of analgesics/drugs for pain management in horses with colic	Number of horses with colic treated with each category of analgesics/drugs (n=375)	Percentages (%) of horses with colic treated with each category of analgesics/drugs
NSAIDs	320	85.3
NSAIDs-opioids	24	6.4
Spasmolytics	22	5.9
NSAIDs-opioids-sedatives	3	0.8
Sedatives	2	0.5
NSAIDs-sedatives	1	0.3
NSAIDs-NSAIDs	1	0.3
Opioids	1	0.3
Opioids-sedatives	1	0.3
Total	375	100

NSAIDS=Nonsteroidal anti-inflammatory drugs

**Table-4:** Frequencies for the use of single and combined analgesics drugs to treat horses with colic as recorded in four Equine Practices from January 2004 to December 2014 in Nairobi County, Kenya.

Single and combinations of analgesics/drugs for treatment of colic	Number of horses with colic treated with each analgesic/drug (n=375)	Percentages (%) of horses treated with each analgesic/drug
Flunixin meglumine	307	81.9
Butorphanol-flunixin meglumine	24	6.4
Spasmolytics	22	5.9
Ketoprofen	13	3.5
Xylazine hydrochloride	2	0.5
Xylazine-butorphanol-flunixin	2	0.5
Xylazine-flunixin	1	0.3
Butorphanol	1	0.3
Ketoprofen-flunixin	1	0.3
Detomidine-butorphanol-flunixin	1	0.3
Butorphanol	1	0.3
Total	375	100

### Gastric intubation and the use of metabolic stimulants in the management of colic

Among the 375 horses with colic, 30.9% (n=116) were intubated as part of the management for the conditions that caused colic. Gastric intubation was done mainly for decompression of gastrointestinal tract in impaction colic and displacement colic. Majority of the horses (69.1%, n=259) were not intubated, of which a large percentage comprised spasmodic colic (n=243), while the rest included impaction colic (n=9), and displacement colic (n=7) as seen in Table-4. All the equine practitioners used mineral oil (liquid paraffin) and water as laxatives, which were introduced into the horse through nasogastric tube.

Metabolic stimulants, mainly vitamin B complex, was used in 22.1% (n=83) of the horses with colic. The remaining 77.9% (n=292) of the horses were treated without any use of metabolic stimulants (Table-4).

### Outcomes of the horses that had colic

The various outcomes of the cases of colic are presented in Tables-5 and 6. In the 11-year period, 94.4% (n=354) of the 375 horses that had colic recovered fully after receiving various forms of treatments, and 5.6% (n=21) died. The deaths occurred due to torsion and volvulus in 57.1% (n=12), impaction colic in 28 % (n=6), RDDLC in 9.5% (n=2), and strangulating lipoma in 4.8% (n=1) of the horses that died. Only 6% (n=23) of the 375 horses were recorded as having had recurrent colic episodes.

**Table-5:** The use of nasogastric intubation and metabolic stimulants for management of various types of colic in horses as recorded by four Equine Practices from January 2004 to December 2014 in Nairobi County, Kenya.

Use of nasogastric intubation and metabolic stimulants	Type of colic			Total

	Spasmodic	Impaction	Intestinal displacement	
Intubated	12	95	9	116
Not intubated	243	9	7	259
Total	255	104	16	375
No metabolic stimulant	235	46	11	292
Used metabolic stimulant	20	58	5	83
Total	255	104	16	375

**Table-6:** Percentages of horses with various types of colic among the 21 that died in the 11-year period as recorded in the four Equine Practices in Nairobi County, Kenya.

Type of colic	Number of horses that died from each type of colic	Percentages (%) of horses that died from each type of colic
Volvulus	12	57.1
Colon Impaction	6	28.6
Right dorsal displacement	2	9.5
Lipoma	1	4.8
Total	21	100

### Association between types of colic and various factors including treatment protocol, recurrence, and outcomes

Simple univariate analysis was performed using Chi-square statistics between types of colic and treatment protocols, recurrence and outcomes of horses with colic. The type of colic that a horse had seemed to significantly (p<0.05) influence the protocol chosen for managing the problem and the chances of recurrence of colic (*χ*^2^=4.6, p = 0.04) (Table-7). The type of colic that a horse had strongly influenced (*χ*^2^=265, p<0.001) the decision on whether or not to perform gastric intubation as part of its management, but weakly influenced (*χ*^2^=4.9, p=0.03) the possibilities of recovery of the horse. Furthermore, strongly associated with the type of colic was the possible final outcome (thus recovery or death) of the horse that suffered the colic (*χ*^2^=250, p<0.001). Type of colic also seemed to strongly (*χ*^2^=99.3, p<0.001) determine the need for the use of metabolic stimulants, particularly vitamin B complex. Other factors that were found to be moderately or weakly associated with the type of colic that a horse had included the analgesia protocol to be used for pain management (*χ*^2^=22.5, p<0.001), whether combined or single analgesic therapy.

**Table-7:** Percentages of horses that had recurrent or non-recurrent colic episodes as well as those that died or recovered among the 375 cases recorded in four Equine Practices in Nairobi County, Kenya.

Nature of occurrence and outcomes of colic cases	Number of horses with each type of colic occurrence and outcome (n=375)	Percentages (%) of horses with each type of colic occurrence and outcome
Recurrent	23	6.1
Non-recurrent	352	93.9
Total	375	100
Died	21	5.6
Recovered	354	94.4
Total	375	100

When these same factors were subjected to multivariate analysis, the only factors that were found to have a negative influence on chances of a horse recovering from colic were non-use of nasogastric intubation, use of metabolic stimulants, and presence of intestinal displacements. These factors had a negative β-estimate and p>0.05 except intestinal displacement. Intestinal displacement (β-estimate=−2.70, odds ratio [OR]=0.1, p=0.007), non-use of nasogastric intubation (β-estimate=−0.049, OR=1.0, p=0.831), and use of metabolic stimulants (β-estimate=−0.006, OR=1.0, p=0.969) (Table-8).

**Table-8:** Association between the type of colic in horses and various factors employed for its management and the outcome that was analyzed from retrospective data recorded from January 2004 to December 2014 in four Equine Practices in Nairobi County, Kenya.

Colic management and outcome factors	Chi-square (χ^2^)	P-value
Gastric intubation	265	<0.001*
Outcome of horse with colic	250	<0.001*
Use of vitamin B complex	99.3	<0.001*
Analgesia protocol	22.5	<0.001*
Combination of analgesics	18.3	<0.001*
Recovery with gastric intubation	4.91	0.03*
Recurrence of colic	4.46	0.04*
Use of NSAIDs	1.74	0.42

* Significant at p<0.05. NSAIDS=Non-steroidal anti-inflammatory drugs

## Discussion

The 3.1% incidence of colic in horses that was found through the retrospective study from the 11-year record in Nairobi County Kenya was also caused by similar forms of colic as in a previous retrospective study of 10-year records in South Africa. The only difference is the study in Kenya records spasmodic colic, while this South African study reports tympanic colic [34]. The incidence compares closely with the 4.2% found in a study of colic cases that occurred over a 1-year period in the United States of America [35]. The low incidence of colic in Nairobi County, Kenya, could probably be attributed to horse owners’ decisions not to report colic to the veterinarians compared to other parts of the world. It has been shown that some horse owners prefer to consult their “mentors” (who are more experienced horse owners) to calling a veterinary surgeon [22]. The order of occurrence of the various types of colic was similar to that reported previously elsewhere with the highest being spasmodic colic, followed by impaction and obstructive or displacement colic [34]. Although obstructive and displacement colic were found to occur at a lower rate in the current study, it has been suggested that presence of a combination of several risk factors could increase the incidence of these types of colic [36].

Since colic can be transient with possibilities of few cases recovering spontaneously as reported previously [37], a number of horse owners are tempted to initially carry out treatment of colic on their own without involving veterinarians. This was a common practice found in the current study. The horse owners resolve to offer first aid using analgesics coupled with walking and lunging the horse [38], and only call the veterinarian if the horse does not respond to these treatments over a period of time.

Pain management and restraint are critical in the treatment of horses with colic. In addition, pain management should be carefully and frequently monitored to evaluate the responsiveness of the treatment [3]. The use of NSAIDs and mostly flunixin meglumine for pain management in the current study agrees with most previous reports showing that this is the drug of choice for management of abdominal pain [39,40]. Flunixin meglumine has been cited as the most appropriate analgesic in equine colic due to the fact that it is the best inhibitor of visceral pain. It has several advantages such as capability to provide analgesia for 8-12 h, control of inflammation and endotoxemia, thus resolving most of the simple medical types of colic [40]. Flunixin meglumine functions by blocking the production of prostaglandins for 8-12 h after a single-dose injection [3]. One other reason for common use of flunixin meglumine is the mere ease of availability. However, its effects in masking pain for colic conditions that may require surgery could be risky for the outcome of the case [39], because after masking of pain, the need for immediate surgery may not be apparent until at a late phase of pathological progression of the disease. In this study, the use of ketoprofen was merely influenced by the availability compared to the commonly available flunixin and not by its efficacy. The choice of NSAID (either flunixin or ketoprofen) did not have influence on the outcome. Another study showed the choice of NSAID did not have major effects on clinical outcomes in horses with strangulating small intestinal lesions but had some effects on signs of pain [41]. Although ketoprofen is an NSAID with similar analgesic effects as flunixin meglumine, it is not widely used for treatment of horses with colic [40]. Similar observations were made about the rare use of ketoprofen in Nairobi County, Kenya. Other studies indicate that commonly used NSAIDs in the treatment of colic are flunixin, followed by phenylbutazone, meloxicam, and ketoprofen in that order [9].

Like in other studies, butorphanol tartrate was found to have been rarely used as single drug in management of colic, and this could possibly be attributed to the fact that it affects intestinal motility when given in high doses, which could have deleterious outcome. It reduces small intestinal motility yet has minimal effect on pelvic flexure activity [39]. However, it was more commonly used in combination with flunixin meglumine. Recent studies have shown that buprenorphine resulted in better post-operative analgesia than butorphanol without causing much physiological disruption than what is normally expected in general anesthesia in horses [42].

The use of buscopan (N-butylscopolammonium bromide) in the current study was found to be confined only to spasmodic colic, which is consistent with the general practice since it is an anticholinergic that provides pain relief through its antispasmodic action as well as a transient decrease in intestinal motility. It relieves abdominal pain by relaxing the smooth muscles, hence its use in spasmodic colic, flatulent colic and impaction colic, but does not provide appreciable relief from colic on its own. It temporarily increases heart rate for up to 30 min following its administration due to its parasympatholytic effects [3,39]. Therefore, antispasmodic drugs indirectly provide analgesia by reducing spasms of the intestine [3]. This study showed that horses that were treated with spasmolytics alone were less likely to recover than those treated with NSAID alone.

In spite of effectiveness of these analgesics in controlling pain, treatment of colic should also be directed toward decompression of the distended stomach or intestine through nasogastric intubation, softening of ingesta by use of laxatives, enteral fluid therapy and restoration of normal gut movements [43].

Majority of horses were not intubated, mainly because most of the cases were spasmodic colic and this positively influenced the outcome of colic. However, the use of nasogastric intubation was mostly in cases of impaction colic, which is consistent with the previous reports that indicated that this was a means of decompression and administration of laxatives as well as water directly into the stomach [18]. Hydration of the colon or administration of substances that increase drawing of water into its lumen such as magnesium sulphate help to soften colonic contents and subsequently resolve the impaction. Similarly, anything that increases motility and oral water intake are helpful in resolving impaction [12]. Administration of vitamin B complex as metabolic stimulant as was done in the horses with impaction colic in this study, helps in energy metabolism and slightly reduces accumulation of lactic acid [44].

The fact that the outcome of colic was associated with the type of colic that a horse had, could be attributed to whether the type of colic responds to medical treatment or requires surgical intervention. This explains the reason for high recovery rate in spasmodic colic than impaction colic or intestinal displacement colic. Spasmodic colic responds well to medical treatment, hence the high rate of recovery as observed in this study. Colic conditions requiring surgery are reported to have a recovery rate that is slightly lower than those needing medical treatment [5,8,33,34].

Other studies showed that large colon volvulus is the most common cause of strangulation obstruction, with strangulating diseases of the small intestine causing the highest mortality rate [36]. The death of all cases of intestinal displacement colic and some of those with impaction colic in the current study can be attributed to the fact that the equine practices in Nairobi County, Kenya, do not perform surgeries for colic. However, some impactions may not resolve for several days, while others will not resolve with medical therapy. Hence, all cases of colic in horses requiring surgery either die or are euthanized. Other studies have shown that the most common reasons for euthanasia included severity of colic, cost of surgery, and non-specific chronic diseases [22,45]. A study conducted in Denmack showed that 59% of all horses recommended for surgery were euthanized, either before surgery or on the operating table, and 10% of the horses were euthanized before the recommended surgical treatment. Euthanasia was necessary in horses that appeared to have poor prognosis and is often a preferred method of alleviating suffering, even though the suffering could also be done with medical or surgical treatment [46]. However, previous reports in other parts of the world suggested that the overall prognosis for the life of a horse and for its return to use after large colon displacement is good [47].

The 23% incidence of recurrent episodes of colic in this study compares closely to 20% in a previous study done in Finland in which horses with small intestinal lesions tended to have longer convalescent time but relatively low incidence of post-operative recurrent colic than horses with large intestinal colic [48]. This previous Finland report indicated that horses operated for large intestinal lesions were 3.3 times more likely to have post-operative colic compared to those operated for small intestinal lesions. Previous reports indicated that approximately 93% of horses treated medically or surgically survived at least up to discharge from the hospital. However, post-operative complications still remain common and impact negatively on horse welfare, survival, return to previous use, and the costs of treatment [49]. Another report indicated that case fatality for some cases may vary from few deaths in cases of simple colic to as high as 75% in colic from strangulated intestine [36].

## Conclusions

The overall incidence of colic in Nairobi County, Kenya, is low, with the most common type being spasmodic colic followed by impaction colic. The most common pain management protocol for colic in horses in Nairobi County, Kenya, is the use of flunixin meglumine, followed by flunixin-butorphanol combination. Surgery for horses with colic in Nairobi County is not commonly done, and therefore, those horses requiring surgery either die or are euthanized.

## Authors’ Contributions

This paper is extracted from a Master of Veterinary Surgery thesis in which AG was the graduate student who collected data and wrote the thesis. JN, VV and EM were supervisors of the research and all participated in designing the project and in the critical process of writing the thesis. Part of the data was provided by VV from his Equine practice, JN assisted in the organization of data analysis and design of the manuscript s in collaboration with AG. EM helped in review of the manuscript. All authors read and approved the final manuscript.
